# Numerical Study on Chemical Kinetic Characteristics of Counterflow Diffusion Flame Extinction of Methane/Ammonia/Air Flame under High Pressure or Air Preheating Temperature

**DOI:** 10.3390/molecules29153632

**Published:** 2024-07-31

**Authors:** Ying Chen, Jingfu Wang, Jian Zhang, Yi Li

**Affiliations:** 1 Beijing Key Laboratory of Control Technology for City Toxic and Combustible Major Hazards, Institute of Urban Safety and Environmental Science, Beijing Academy of Science and Technology, Beijing 100050, China; yingchen0511@outlook.com; 2 MOE Key Laboratory of Enhanced Heat Transfer and Energy Conservation, Beijing University of Technology, Beijing 100124, China; zhangjian123@emails.bjut.edu.cn (J.Z.); liyi982021@163.com (Y.L.); 3 Beijing Key Laboratory of Heat Transfer and Energy Conversion, Beijing University of Technology, Beijing 100124, China

**Keywords:** methane/ammonia mixed fuel, counterflow diffusion flame, chemical kinetic characteristics, extinction limits

## Abstract

Green ammonia has become an increasingly popular fuel in recent years because of its combustion process without carbon oxide release. Adding ammonia to methane fuel for co-combustion has become one of the important research topics in the current combustion field. In the present study, the CH_4_/NH_3_/Air counterflow diffusion flame was taken as the research object, and Chemkin-2019 R3 software was used to explore and analyze the flame extinction limit and chemical kinetics characteristics under different ammonia mixing ratios, initial pressures, and air preheating temperatures. It was obtained that the flame extinction stretch rate was decreased by increasing the NH_3_ mole fraction in the CH_4_/NH_3_ mixed fuel. The increase in pressure or air preheating temperature would accelerate the chemical reaction rate of each component in the combustion process, increase the flame extinction limit, and counteract the “stretching effect” of the flame, thus restraining the flame extinguishing phenomenon. The results of a path analysis show that the formation and consumption of OH had an important influence on flame extinction in the chain reaction. The net reaction rate of OH increases with increasing the initial pressure or air preheating temperature, which leads to an increase in flame intensity, combustion stability, and the extinction limit. Furthermore, the function curve between the reaction influences the RIF factor and the stretch rate of the first-to-ten reactions, affected by the heat release of flame combustion, was drawn and quantitatively analyzed. Eventually, a sensitivity analysis of the flame under different working conditions was completed, which found that promoting the forward reaction R39 H + O_2_<=>O + OH also promotes the positive combustion as a whole when the flame was near extinction. The sensitivity coefficient of R39 in the CH_4_/NH_3_/Air flame increases with the growing initial pressure. The increasing air preheating temperature was capable of switching the reaction of R248 NH_2_ + OH<=>NH + H_2_O in the CH_4_/NH_3_/Air flame from an inhibiting reaction to a promoting reaction, while decreasing the sensitivity coefficient of inhibiting the forward reaction R10 O + CH_3_<=>H + CH_2_O, R88 OH + HO_2_<=>O_2_ + H_2_O, and R271 H + NO + M<=>HNO + M. Thus, the inhibition effect of flame extinction was weakened, and the positive progress of combustion was promoted.

## 1. Introduction

As a kind of nitrogen–hydrogen compound, ammonia energy has already attracted plenty of attention all over the world, and is set to become an important hydrogen-based fuel in light of its background in carbon neutralization, owing to its unique physical and chemical properties, which have the advantages of convenient storage and transportation, zero carbon emission, and high thermal efficiency. Among all the countries interested in ammonia, China proposed the energy strategic goal at the beginning of 2020 for achieving “Carbon Emission Peak” by 2030 and “Carbon Neutrality” by 2060 [1]. By the end of 2019, the Shanghai Shipbuilding Research and Design Institute (SDARI) under the China Shipbuilding Industry Corporation had realized the demand for zero carbon emission of the main engine on a 180,000 t ammonia fuel ship [2]. In 2022, the 50,000 t ammonia fuel-powered medium-sized oil tanker/chemical tanker (MR), which was independently developed and designed by SDARI, obtained the AIP certificate granted by Registro Italiano Navale (RINA), and achieved the latest achievements in green, environmental protection, and zero carbon fields as well. Japan regards ammonia as an essential alternative fuel to achieve an 80% reduction in greenhouse gas emissions by 2050 [3], and it was first introduced into “The Strategic Energy Plan of Japan” in 2021; it aims to be used as a combustion fuel for power generation after being mixed with natural gas or pulverized coal [4]. In early 2020, the Malaysia International Shipping Corporation, Samsung Heavy Industry of South Korea, Lloyd Shipping Society of the United Kingdom, and the German marine manufacturer Man Energy Company reached a cooperative intention to jointly develop domestic ammonia fuel cruise ships in the next 3–4 years [5]. With the rapid development of science and technology, the use technology of ammonia fuel has gradually matured and is being vigorously promoted in the civil field. It will be able to achieve the efficient combustion of ammonia fuel, which will be widely used in aviation engines, civil automotive engines, gas turbines, boilers, and fuel cells in the future. Therefore, as a new type of secondary energy source, ammonia fuel has superb development potential, market prospects, and research value.

Although the application of ammonia fuel has predictable benefits, it can only be realized after scholars have an accurate understanding of its combustion characteristics. In recent years, most scholars have carried out a series of experimental or numerical studies on the combustion characteristics of ammonia fuel and ammonia mixed fuel, including ignition delay time [6,7,8,9], laminar flame propagation speed [10,11,12,13,14], Markstein length [10,13,15], and extinction limit [16,17,18]. Xiao et al. [16] evaluated computationally, utilizing counterflow premixed ammonia/methane/air flames at normal temperature and pressure conditions, which shows that methane addition substantially increases the flame sustainability to a high stretch rate. Colson et al. [17] studied the ammonia flame extinction stretch rate both experimentally and numerically and investigated the effects of pressure on its extinction characteristics. The results show that the increase in extinction stretch rate with pressure was greater in the case of ammonia/air flame. And, they found that the different rates of dependence on pressure of the reaction path of the two fuels could explain this phenomenon. Zhang et al. [18] numerically studied the extinction stretch rate of the NH_3_/CH_4_-air laminar premixed flame at a normal temperature and pressure (NTP), which revealed that the increasing CH_4_ mole fraction in the NH_3_/CH_4_ mixed fuel promotes the flame extinction stretch rate. The design of the combustion chamber in the ideal state should satisfy the stable combustion of the flame under the widest possible conditions, and the flame cannot be extinguished due to the change of boundary conditions and chemical kinetic factors of combustion (such as the reaction rate of active component, activation energy, and chain reaction rate); thus, it is necessary to study the chemical kinetic characteristics of flame extinction. However, most scholars have studied the kinetic characteristics of chemical reactions focused on alkane as the main fuel [19,20,21,22,23], while there are extremely few studies on the chemical kinetic characteristics near flame extinction of ammonia fuel. A great number of studies related to ammonia is based on the development of new chemical kinetic models for numerical or experimental studies on combustion characteristics. For instance, Colson et al. [17] accomplished experiments with counterflow flame burners and numerically simulated the stretching effect of NH_3_/Air premixed flames. Then, the change of the flame extinction limit under different equivalence ratios and different initial pressures is discussed, and the reaction path of the NH_3_/Air flame and CH_4_/Air flame is explored. Otomo et al. [24] developed an improved ammoxidation chemical kinetic modeling method based on the Song mechanism [25], which revealed the reported values of laminar flame velocity and ignition delay time of NH_3_/H_2_/Air in a considerable equivalent ratio and pressure range. In the latest research results, Chen et al. [26] developed a detailed chemical reaction mechanism suitable for the combustion of NH_3_/dimethyl mixed fuel by experiments and a numerical study. The extinction limits of the diffusion flames of the NH_3_/dimethyl mixture were obtained. It was found that the chemical and thermal effects of DME promote the combustion of the NH_3_-DME mixture, while the transport effect inhibits this, and the promoting effect of chemical effect increases with the increasing DME mole fraction.

Therefore, there is an extreme urgency to study the chemical kinetics characteristics of ammonia-related fuels for the practical engineering application of ammonia. In particular, the research on the chemical kinetics of methane/ammonia mixed fuel has heretofore been lacking in this field. Based on this background knowledge, the extinction limit and chemical reaction kinetic characteristics of flame extinguishing under different ammonia blending ratios, initial pressures, and initial temperatures of the oxidant side of the CH_4_/NH_3_ mixed fuel and air diffusion combustion are studied by a counterflow flame method in the present study. Our research content has a certain guiding significance for the practical application of CH_4_/NH_3_ fuel or pure ammonia fuel in combustion equipment.

## 2. Results and Discussions

### 2.1. Extinction Limits

**Figure 1:**
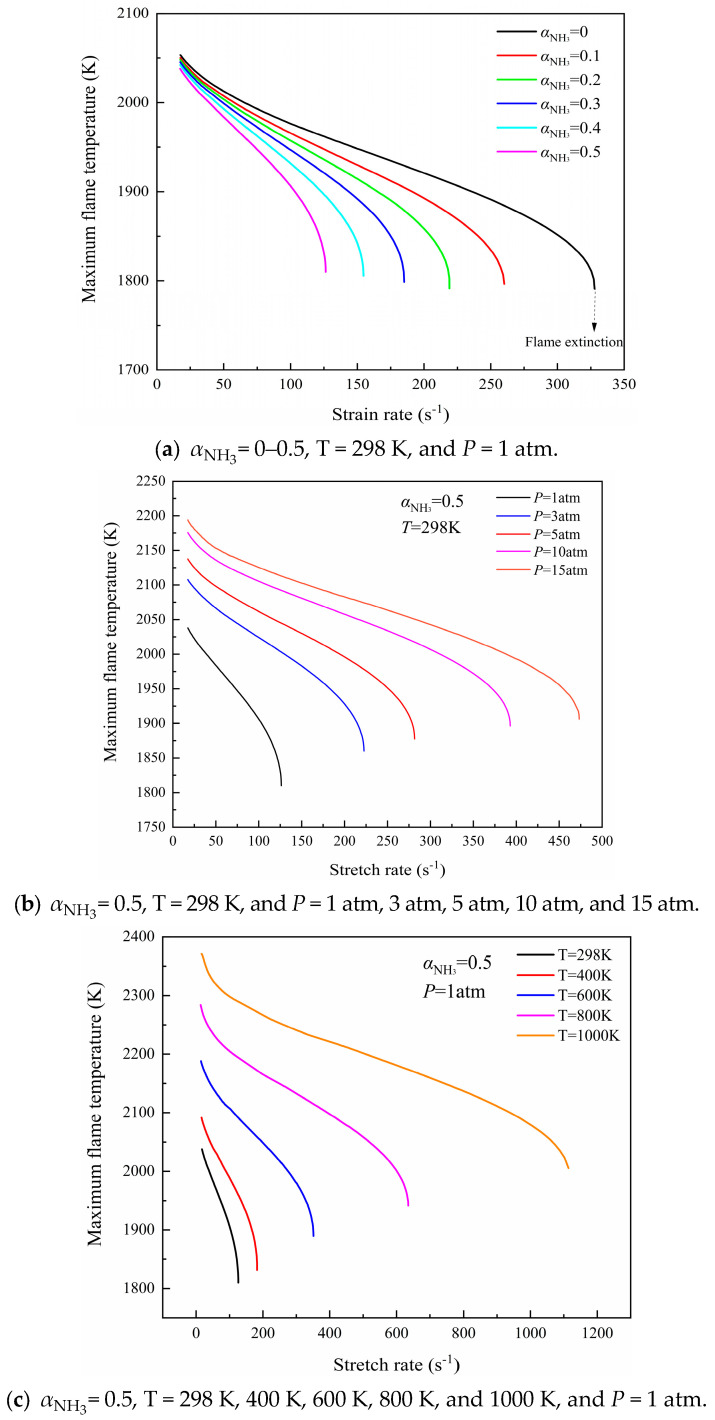
Computed maximum flame temperature of CH_4_/NH_3_ versus air counterflow diffusion flames with different conditions.

The maximum flame temperature, as a function of stretch rate, can effectively reflect the difference in flame intensity when the flame is extinguished in different working conditions [27]. The computational maximum flame temperature versus the stretch rate at ammonia blending ratios of 0~0.5, initial pressures of 1~15 atm, and oxidizer temperatures of 298 K~1000 K are shown in Figure 1. It can be seen from Figure 1a that the increase in the ammonia blending ratio is able to reduce the flame extinction limit and the maximum flame temperature. Figure 1b,c show that the increase in initial pressure or oxidizer side temperature can significantly widen the flame extinction limit and enhance the combustion stability of the flame. Moreover, the maximum flame temperature under different working conditions decreases gradually with the increase in stretch rate, which is mainly due to the shorter stagnation residence time of the gas component reaction and the thinning of flame thickness caused by the stretching effect of the flame. Then, the chemical reaction rate is limited, the combustion heat production decreases, and the convective heat loss increases; thus, finally the flame is extinguished. The calculated maximum flame temperature increases obviously with the increase in the initial pressure at the same stretch rate, and the flammable range of the flame is broadened as well. The chemical reaction rate of the components grows rapidly as a result of flame compression, intensifying the collision between gas molecules in the reaction area.

It is revealed by numerical results that the increase in initial pressure can increase the flame combustion peak temperature, accelerate the chemical reaction rate, and increase the heat release, so as to offset the adverse influence of the “stretching effect” on the flame, and then restrain the flame extinguishing phenomenon, so that the flame extinction limit becomes larger.

The increase in air preheating temperature can effectively solve the problem of energy imbalance between fluid heat convection heat transfer and combustion chemical reaction heat release on both sides of the fuel and oxidizer due to the flame stretching effect. According to the Arrhenius equation and molecular dynamics theory, the chemical reaction rate will increase with the increase in air preheating temperature; therefore, the number of activated molecules in the combustion reaction zone and the number of collisions between them will surge expeditiously, while the reaction rate of each component will increase. Then, the deviation degree of each combustion reaction will decrease, and the combustion environment will be improved.

**Figure 2:**
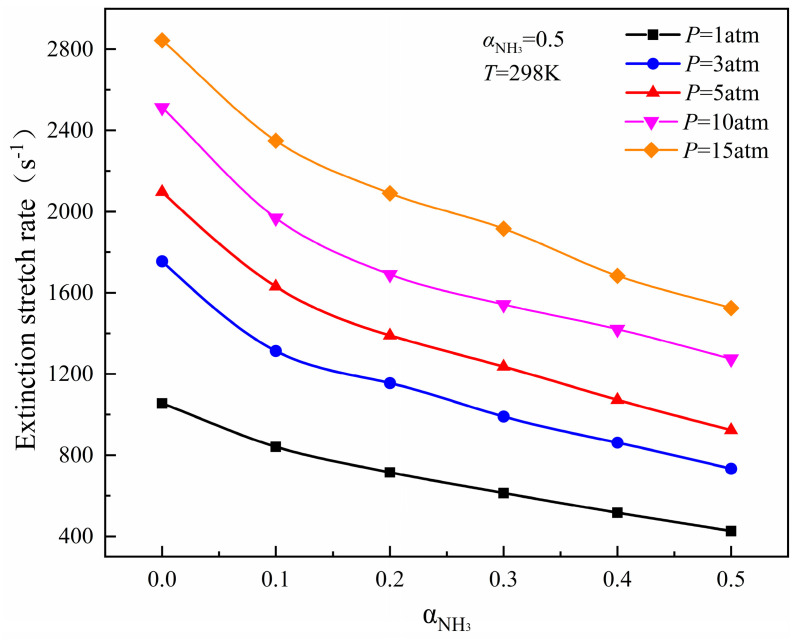
Variation trend of flame extinction stretch rates with ammonia blending ratios under different initial pressure environments.

**Figure 3:**
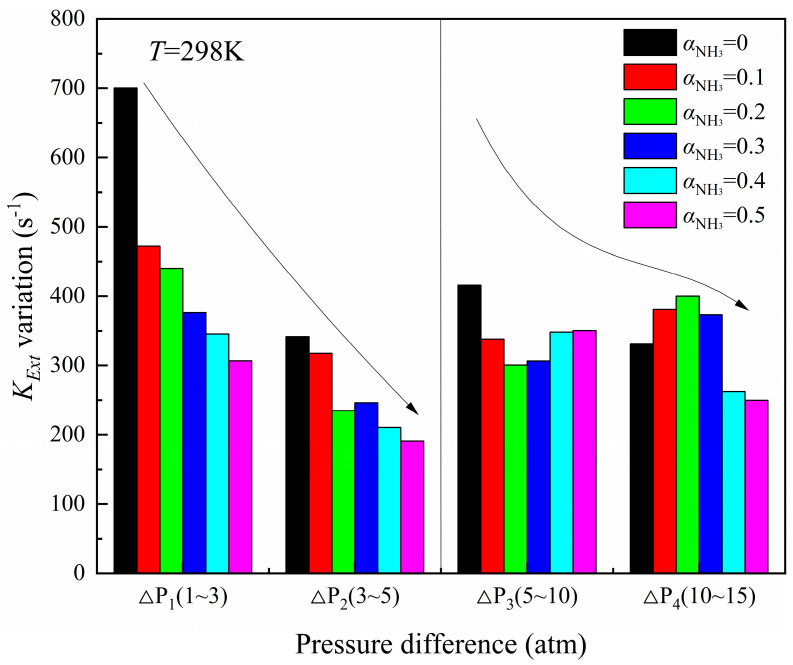
Variation of extinction stretch rates under different pressure differences.

Figure 2 and Figure 3, respectively, show the computation results of the extinction stretch rate as a function of ammonia blending ratios under different initial pressures and the variation of flame extinction stretch rate under different pressure differences. Whether it is pure methane fuel or methane/ammonia mixed fuel, the flame extinction stretch rate always rises with the increase in initial pressure, but the increment of *K_Ext_* is inconsistent under different pressures.

In the range of 1–5 atm, for the six kinds of flames with ammonia blending ratio in the range of 0~0.5, the increase in the flame *K_Ext_* decreases significantly with each increase of 2 atm, and in the range of 3~5 atm, the flame *K_Ext_* increment with ammonia blending ratio 0.3 increases compared with 0.2. In the range of 5~15 atm, the flame *K_Ext_* increment with different ammonia blending ratios shows a disorderly change. When the ammonia mixing ratio of 0.1, 0.2, and 0.3 is increased by 5 atm, the *K_Ext_* increment of the three kinds of flame increases, while the *K_Ext_* increment of the other three kinds of flame decreases. This phenomenon may be caused by the mixing of NH_3_ under high pressures, changing the enthalpy flux at the fuel side, and the thermodynamic effect of NH_3_ strengthens and weakens the flame extinction limit from time to time. The reaction rate of each element fluctuates greatly under high pressure. It can be found from Figure 3 that 5 atm is the critical pressure at the beginning of large variations and fluctuations. When the pressure is greater than 5 atm, ammonia undergoes a phase transition reaction from the gas phase to the liquid phase, while releasing an amount of heat. The extreme instability of the combustion process and the complexity of flame extinction characteristics results in the poor adaptability of the Okafor mechanism to flame prediction. Therefore, further experimental research on the extinction characteristics of the CH_4_/NH_3_/Air diffusion flame with high pressure is needed to improve the adaptability of the mechanism for the ammonia flame under high pressure.

**Figure 4:**
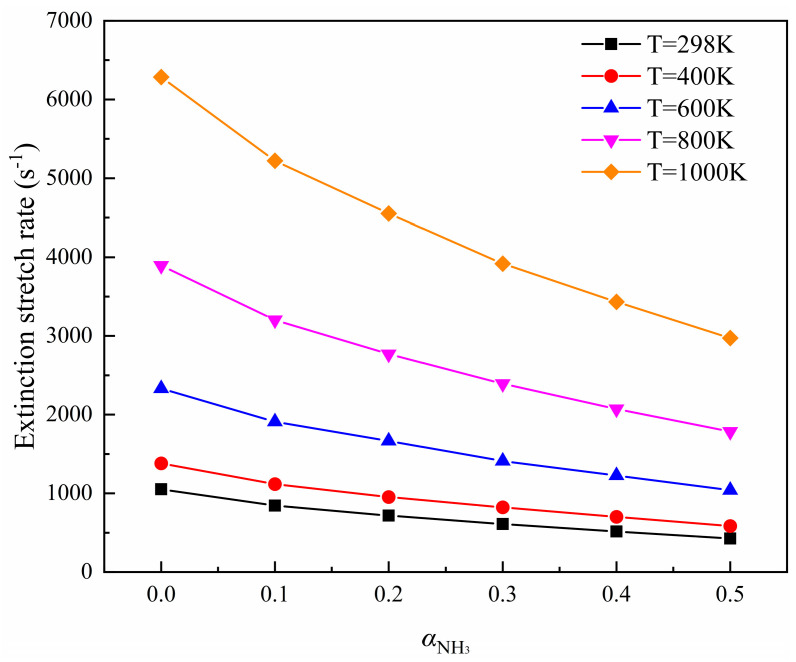
Variation trend of flame extinction stretch rates with ammonia blending ratios at different air preheating temperatures.

**Figure 5:**
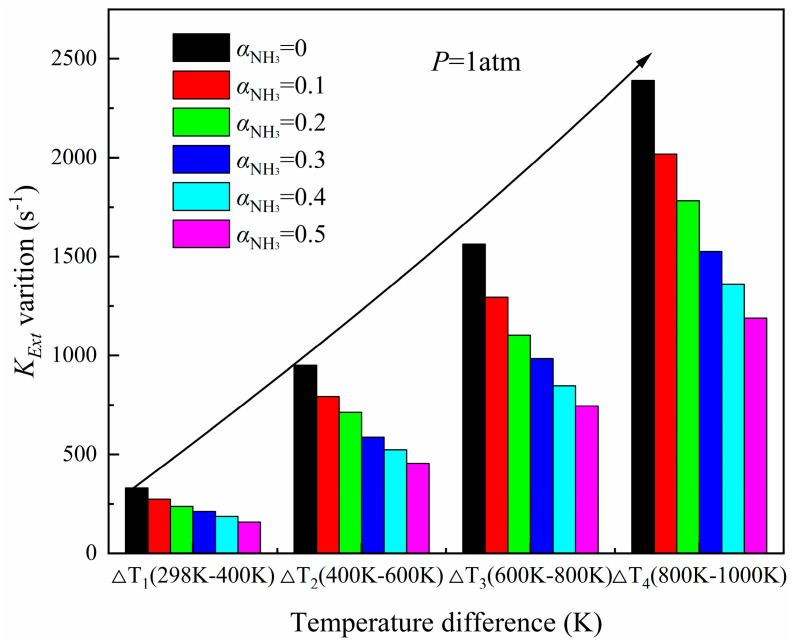
Variation of extinction stretch rates under different temperature differences.

**Figure 6:**
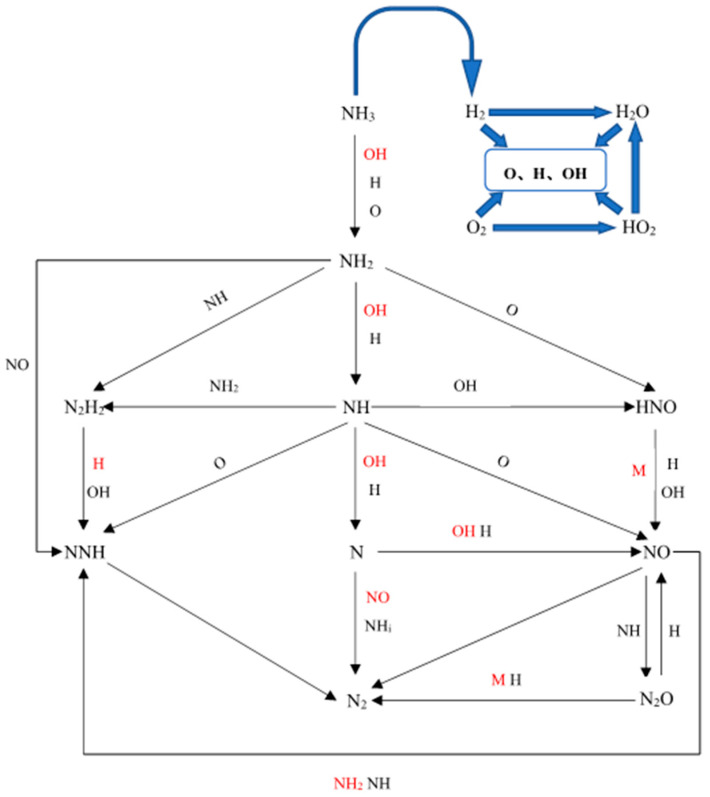
Reaction path of NH_3_ in fuel combustion.

Figure 4 shows the variation trend of flame extinction stretch rates with an ammonia blending ratio at different air preheating temperatures. Figure 5 shows the variation of the flame extinction stretch rate with temperature differences at different ammonia blending ratios. As illustrated and demonstrated in Figure 4, an increase in the air preheat temperature leads to an improvement in the flame extinction stretch rate. In Figure 6, the *K_Ext_* variation at different temperature differences decreases with the increase in the ammonia blending ratio, and the larger the air preheat temperature, the greater the decrease, which indicates that ammonia addition will lower the flame extinction limit and make combustion more difficult.

In the range of 298 K–1000 K, the rising range of *K_Ext_* increases gradually with a total increase of 200 K, which firmly proves that increasing the air preheating temperature can effectively increase the flow stability of incoming gas in the process of flame combustion, and can balance the adverse effects of flame instability caused by the addition of NH_3_. Then, the flame can burn more stably.

### 2.2. Reaction Path of NH_3_ in Fuel Combustion

Based on the path analysis method, the NH_3_/Air counterflow diffusion flame and the CH_4_/NH_3_/Air counterflow diffusion flame with a 50% ammonia mixture are studied, and we attempt to discuss the chemical kinetic characteristics of mixed fuel during diffusion combustion. The flame stretching effect mainly affects the flame thickness, flame propagation velocity (which mainly affects the component diffusion coefficient in the diffusion flame), and chemical reaction rate with the increase in stretch rate in the process of NH_3_ burning from ignition to near extinction. However, it makes no attempt to change much on the shunt form of the NH_3_ oxidation path; merely, the contribution ratio of each component to the superior reaction is transformed. There are three main oxidation paths of ammonia, which are as follows: (1) the dehydrogenation of NH_3_, (2) the oxidation of NH*_i_* (*i* = 0, 1, 2), and (3) the polymerization of NH*_i_* + NH*_j_* (*i*, *j* = 0, 1, 2). The combustion chemical reaction path of NH_3_ in the oxidation process when the flame is near extinction is obtained in this study, as shown in Figure 6. The red characters represent the radicals contributing more in the single path, and the blue clipping head and the square part represent the active radical pool of the H_2_-O_2_ system in the combustion reaction.

A large number of active radicals (i.e., O, H, and OH) in the system diffuse from the high-temperature combustion zone to the low-temperature combustion zone, and participate in each step of the reaction after the fuel is ignited. The existence of these active radicals is conducive to the combustion cycle itself and the conversion of flame energy. Subsequently, the shunt analysis of the chain reaction shows that three main chain branches can be observed from the amino group; the oxidation process of the main chain (N1 chain) NH_3_→NH_2_→NH→N is called the dehydrogenation reaction of NH_3_, which is similar to the oxidation process of the CH_4_/Air counterflow diffusion flame [17]. The chain initiation reaction is through the dehydrogenation reaction of H, O, OH, and other active radicals (i.e., OH) to form amino (i.e., NH_2_) or methyl (i.e., CH_3_).

In the oxidation of ammonia, the value of the ratio of the O radical to the H radical concentration has a great influence on the oxidation process of NH_3_ dehydrogenation derivatives; the diffusion flame is lean burn when the O radical concentration is greater than H radical concentration, which is shown in the branched chain (N_2_ chain) NH_3_→NH_2_→HNO→NO→N_2_O→N_2_, and the NH_i_ group can form NO through the intermediate HNO. Finally, most of the oxidation reactions of HNO to NO need to go through a three-body M collision and absorb a large amount of heat from the flame, especially when the stretch rate gradually increases to the near-extinguished state, thus destroying the heat balance of the flame, which may lead to flame extinguishment.

**Figure 7:**
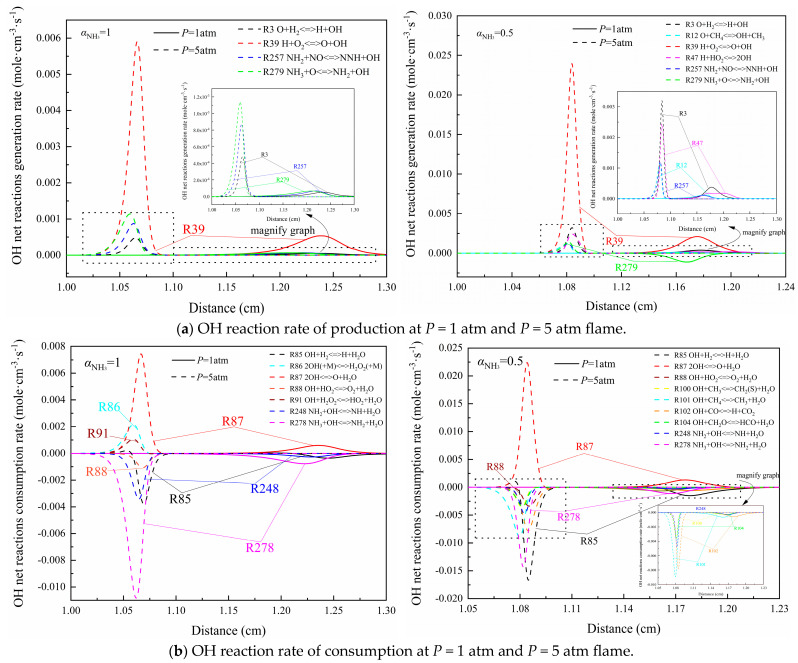
Generation and consumption rates of OH radicals in two kinds of flame near extinction state under different initial pressures.

**Figure 8:**
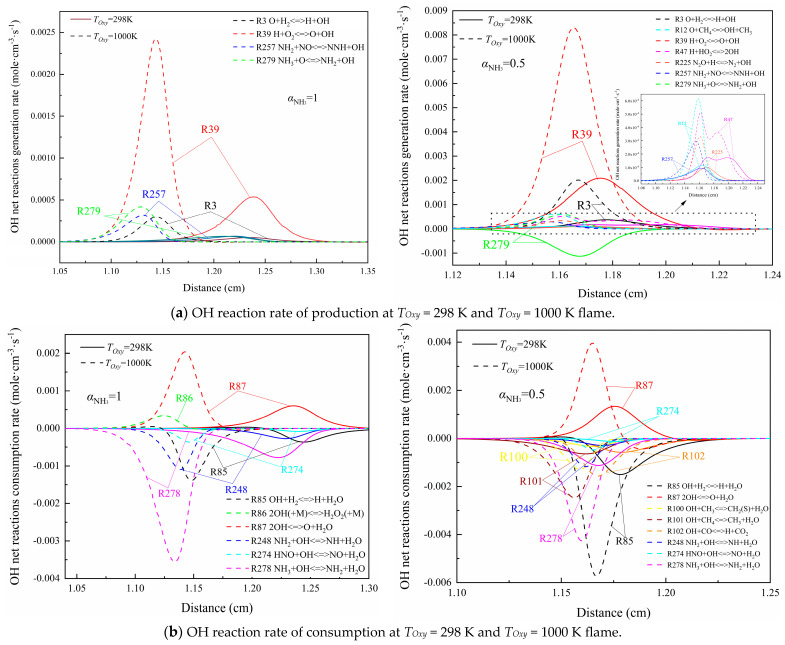
Generation and consumption rates of OH radicals in two kinds of flame near extinction state at different air preheating temperatures.

In the analysis of combustion chemical kinetics, the alterations of O, H, and OH radicals are extremely important to the whole chemical path analysis process, which directly affects the reaction path of the combustion chemical reaction process [28]. The molar formation rate of the OH radical is defined in ref. [29], representing the overall reaction rate of the flame and the peak position of the mole fraction represents the position of the flame front, while the chemical reaction intensity can be represented by the net reaction rate of the chain branch of OH. The computation results of the OH reaction rates of two counterflow diffusion flames (the NH_3_/Air flame and the CH_4_/NH_3_/Air flame with an ammonia blending ratio of 0.5) in each reaction at different initial pressures or initial temperatures on the oxidant side are depicted in Figure 7 and Figure 8.

Figure 7a shows the variation of the net generation rate of OH under different pressures. It can be found that the OH generation rate of each reaction increases significantly after increasing the pressure. Taking the fastest reaction R39 H + O_2_<=>O + OH into account, the computation results of the OH net generation rate and extinction stretch rate of the NH_3_/Air flame increased about five times (from 1.65 × 10^−4^ mole·cm^−3^·s^−1^ to 8.23 × 10^−4^ mole·cm^−3^·s^−1^) and three times (from 91.93 s^−1^ to 296.64 s^−1^), respectively, while those of the CH_4_/NH_3_/Air flame increased about ten times (from 6.26 × 10^−4^ mole·cm^−3^·s^−1^ to 6.47 × 10^−3^ mole·cm^−3^·s^−1^) and two times (from 417.63 s^−1^ to 919.34 s^−1^), respectively, from 1 atm to 5 atm. Meanwhile, R47 H + HO_2_<=>2OH has two equal peaks of OH production rate similar to “camel”, which becomes one peak with the increase in initial pressure. The increase in pressure may cause the two peaks to be “oppressed” to a certain extent, thus forming a single peak.

An analysis from the perspective of OH radical consumption is shown in Figure 7b. It is clear that R278 NH_3_ + OH<=>NH_2_ + H_2_O has always made the greatest contribution to the consumption of OH in the NH_3_/Air flame. After a comprehensive analysis, the HO_2_ radical would be activated through R88 OH + HO_2_<=>O_2_ + H_2_O and R91 OH + H_2_O_2_<=>HO_2_ + H_2_O with the increase in pressure, which could energetically participate in the cycle between active radicals in the follow-up reaction. In the NH_3_/CH_4_/Air flame with an ammonia blending ratio of 0.5, the change of initial pressure not only continuously keeps the status of R85, but also still contributes the most to the consumption of OH. In the meantime, the consumption rates of OH in other reactions of forward compound H_2_O formation such as R100 OH + CH_3_<=>CH_2_(S) + H_2_O, R101 OH + CH_4_<=>CH_3_ + H_2_O, R102 OH + CO<=>H + CO_2_, R104 OH + CH_2_O<=>HCO + H_2_O, R248 NH_2_ + OH<=>NH + H_2_O, and R278 increase in different degrees, and the peak positions all move to the fuel side.

In conclusion, the net reaction rate of OH is greatly accelerated and the time to reach the peak becomes less when the initial pressure is increased, which causes the growth of flame intensity and the broadening of the extinction limit.

Compared with the effect of the increase in pressure on the branching reaction rate of the OH chain, the increase in air preheating temperature also has a similar effect on it, as shown in Figure 8a,b. R39 is the reaction that contributes the most to the formation of OH radicals from the beginning to end, and R278 is the reaction that contributes the most to the consumption of OH. It is worth noting that R279 NH_3_ + O<=>NH_2_ + OH changes from the reverse reaction in the *T_Oxy_* = 298 K flame to the positive reaction in the *T_Oxy_* = 1000 K flame in the combustion of the CH_4_/NH_3_ mixed fuel. Relatively speaking, it will increase the production of OH free radicals and promote the circulation of reactions in the active free radical pool, playing an essential role in maintaining the stability of the flame.

In the pure ammonia flame with an air preheating temperature of 1000 K, R86 2OH(+M)<=>H_2_O_2_(+M) belongs to the reaction of reverse generation of OH, and the decomposition of H_2_O_2_ is able to control the reaction activity of the high-temperature reaction system. H_2_O_2_ in R86 collides with three-body M (mainly including H_2_O and H_2_) to absorb a large amount of heat in the flame, thus damaging the heat balance of the flame to a certain extent. Although this reaction contributes to the generation of OH, it is unfavorable for the stability of flame combustion and has a certain role in promoting flame extinction.

### 2.3. The Effect of Ammonia Blending Ratio, Pressure, and Oxidant Side Temperature on RIF

In order to further study the influence of chemical kinetics on the counterflow diffusion flame extinction process, the reaction with top-ten reactions forwards the heat release rate in the process of the flame extinction of the NH_3_/Air flame, and CH_4_/NH_3_/Air with an ammonia blending ratio of 0.5 is taken as the research object. The selected results are shown in Table 1 and Table 2 and the pre-exponential factor *A* of each reaction is shown in the table as well. The dimensionless constant of “Reaction Impact Factor”(RIF) is introduced to deeply study the trend of these reactions in the whole flame extinction process, which can be precisely defined by the following equation [30]:

(1) $$\mathrm{RIF}=\int_{-\infty }^{+\infty }\left|q_{i}\right|dy/\sum_{i}^{k}\int_{-\infty }^{+\infty }\left|q_{i}\right|\;dy$$

where *q_i_* is the net reaction rate of the reaction of *i* and k is the total reaction number (there are 356 reactions in the Okafor mechanism). For the purpose of eliminating the influence of forward reaction and reverse reaction, the calculated net reaction rate is taken as an absolute value and then integrated into the whole calculation domain (0–2 cm) to observe the reaction intensity of each elementary reaction in the whole combustion process.

**Figure 9:**
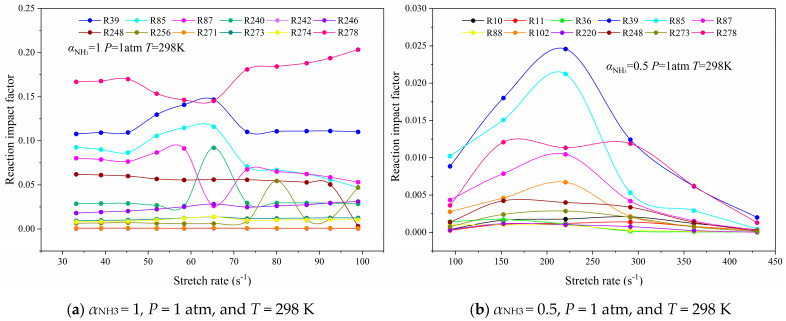
Curves of the reaction impact factor of the NH_3_/Air flame and the CH_4_/NH_3_/Air flame as a function of the stretch rate.

The RIF computation results of two kinds of flame are as shown in Figure 9. As we can see in Figure 9a, the RIF of R278 in the NH_3_/Air flame is the largest, which decreases at first and then increases with the increase in stretch rate. The RIF of R39 is second only to R278, and even the RIF between the elongation 60 s^−1^~70 s^−1^ is higher than that of R278. In this range, the formation rate of OH is accelerated and the flame intensity is improved. R39 is a crucial chain-branching reaction in the vigorous radical pool. A large number of OH and O radicals is generated after the collision of the H atom with O_2_, which makes a great contribution to the formation of the active radical pool. And, it can satisfy the oxidation supply of the NH_i_ group in the initial dehydrogenation reaction. Furthermore, it can be observed that the RIF variation curve of R85 OH + H_2_<=>H + H_2_O is basically the same as that of R39; however, the RIF of R85 is lower than that of R39, especially as the difference is even larger when it is near the extinction limit. In addition, the RIF of R87 2OH<=>O + H_2_O, R240 NH + NO<=>N_2_O + H, and R256 NH_2_ + NO<=>N_2_ + H_2_O fluctuates considerably when the flame is about to extinguish and the RIF is decreasing as a whole in the process of flame extinction. Among this, R240 and R256 are the intermediate steps for the reaction of the NH_i_ groups with NO to form N_2_ and H_2_O, which can reduce a certain amount of NO and be beneficial to combustion.

Figure 9b exhibits the RIF calculation results of the CH_4_/NH_3_/Air counterflow diffusion flame. What can be seen in Figure 9b is that the RIF of R39 completely exceeds that of R278, and even the RIF of R85 also exceeds R278 in the range of the middle and low stretch rates. Moreover, the RIF of R39 and R85 shows a parabolic trend, which is more obvious than that of the NH_3_/air flame, but the overall RIF value is lower than that of the NH_3_/air flame. The RIF of the reactions that contribute substantially to the heat release of the flame decreases obviously due to the addition of CH_4_, which indicates that the chemical reaction dynamics have less influence on the combustion stability of the flame, and the flame can better withstand the adverse effects of the stretching effect.

**Figure 10:**
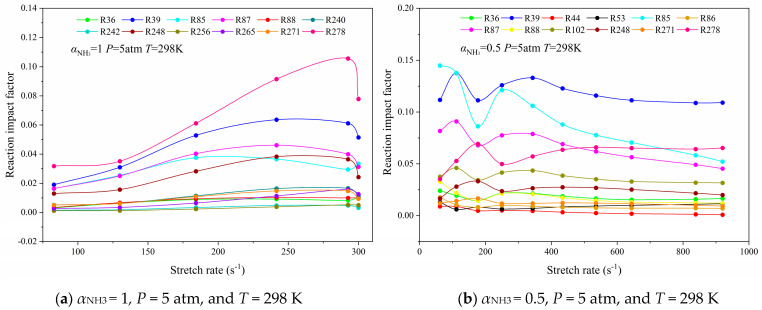
Curves of the reaction impact factor of the NH_3_/Air flame with *P* = 5 atm and the NH_3_/CH_4_/Air flame as a function of the stretch rate.

**Figure 11:**
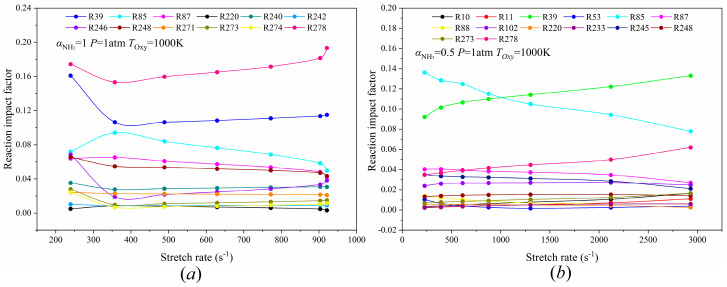
(**a**) α_NH3_ = 1, *P* = 1 atm, and *T_Oxy_* = 1000 K. (**b**) α_NH3_ = 0.5, *P* = 1 atm, and *T_Oxy_* = 1000 K. Curves of the reaction impact factor of the NH_3_/Air flame and the NH_3_/CH_4_/Air flame as a function of the stretch rate at *T_Oxy_* = 1000 K.

The chemical kinetics of flame combustion converts with the increase in initial pressure or air preheating temperature. Therefore, the reaction that contributes significantly to the flame heat release rate in the whole combustion process under two simulation conditions is also selected as the object of study, as presented in Table 3, Table 4, Table 5 and Table 6. Then, the RIF of two kinds of flame in *P* = 5 atm and *T*_Oxy_ = 1000 K is depicted in Figure 10 and Figure 11, respectively.

As can be seen from Figure 10a, the increase in pressure remains still with the status of R278, but brings about the RIF alteration trend showing relatively stable growth, and the chain propagation rate accelerated. In respect to R39, R85 and R87 are essential chain-branching reactions in the active radical pool and have the most major contribution to the formation of OH; the RIF of which would start to decrease near flame extinction with the increase in pressure. The decrease in RIF will lead to the deficiency of active radicals, thus weakening the reaction intensity of the “N1” and “N2” chain reactions, and then the flame intensity is reduced and the flame is extinguished.

As can be deduced from Figure 10b, the RIF of each reaction of the CH_4_/NH_3_/Air counterflow diffusion flame at *P* = 5 atm fluctuates enormously at a low stretch rate, but the fluctuation is no longer obvious when the stretch rate exceeds 400 s^−1^. The RIF of *P* = 5 atm flame is significantly higher than that of the normal pressure flame, which means that the reaction is currently more intense. In addition, R278 is different from other reactions in which the value of RIF increases slowly when it is close to flame extinction, indicating that the increase in pressure can promote the combustion of NH_3_ in the mixed fuel, and the combustion can be more complete when the flame is closer to the extinguished state.

As mentioned in Figure 11a, the fluctuation of flame RIF at high air preheating temperatures is small on the whole, and the RIF curve of the majority of reactions is similar to a straight line. The RIF of R278 in the NH_3_/Air counterflow diffusion flame of *T_Oxy_* = 1000 K is continuously the largest and increases with the increase in stretch rate, and the changing trend of R39 and R85 RIF is no longer consistent. The change of RIF in endothermic reactions R39 and R278, and the exothermic reaction R85, will cause the flame to absorb more heat, resulting in the continuous decrease in the maximum temperature of the flame, the weakening of the flame intensity, and the extinguishment of the flame.

On the other hand, the curves of R39 and R85, as RIF changes with the stretch rate, present a cross distribution in the CH_4_/NH_3_/Air counterflow diffusion flame with *T_Oxy_* = 1000 K and an ammonia blending rate of 0.5, in which the stretch rate corresponding to the intersection point is about 1000 s^−1^, as shown in Figure 11b. Coincidentally, the stretch rate of 1000 s^−1^ is in proximity to the extinction stretch rate of the NH_3_/Air flame at the air preheating temperature of 1000 K, which might be one of the potential reasons for the intersection of the two curves. Generally speaking, the RIF curve of the reaction of the CH_4_/NH_3_/Air flame is no longer as disorganized as in Figure 9b, but it tends to be stable as a whole with the increase in air preheating temperature, which means that the flame combustion stability is improved and the extinction limit becomes larger.

### 2.4. Sensitivity Analysis of Flame Extinction

For the purpose of further exploring whether the selected reaction in the NH_3_/Air and the CH_4_/NH_3_/Air flame could promote or inhibit the flame extinction limits, and the extent of this effect under different working conditions (*P* = 5 atm and *T_Oxy_* = 1000 K), it is necessary to conduct a sensitivity analysis on every single prominent reaction in which main heat is supplied to the combustion system. The sensitivity analysis method for the flame extinction limit is used to increase or decrease the pre-exponential factor *A* of the selected reaction with a high heat release rate by two times, respectively, according to the detailed chemical reaction mechanism, and is used to take the logarithm to ultimately find the quotient. The sensitivity coefficient can be expressed by Equation (2):

(2) $$S=\frac{\ln(K_{Ext^{+}}/K_{Ext^{-}})}{\ln(A_{+}/A_{-})}=\frac{\ln(K_{Ext^{+}}/K_{Ext^{-}})}{\ln(2/0.5)}$$

where *K_Ext_^+^* and *K_Ext_^−^* are the flame extinction stretch rates calculated after the pre-exponential factor corresponding to the selected reaction increases or decreases by two times, respectively. *A*_+_ and *A*_−_ represent the pre-exponential factors after the selected reaction is increased or decreased by two times, respectively. The computation results of the sensitivity coefficient *S* are positive, which means that the reaction plays a role in promoting combustion when the flame is near the extinction limit (hereinafter called “promoting reaction”). On the contrary, it means that the reaction is a reaction promoting flame extinction when *S* values are negative (hereinafter called “inhibiting reaction”).

**Figure 12:**
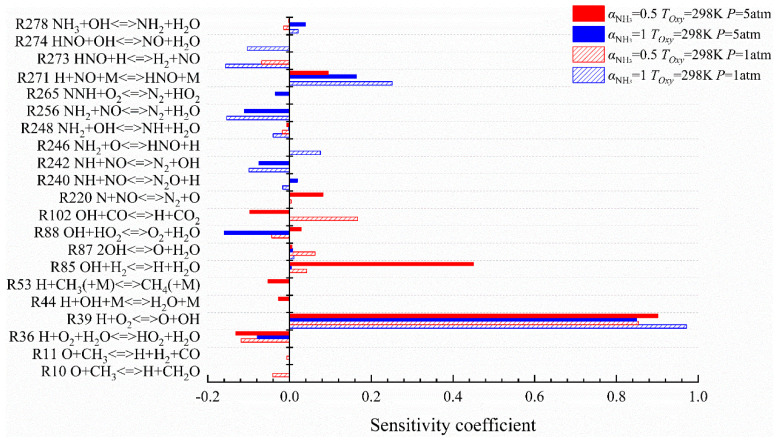
Corresponding reaction sensitivity coefficients of two kinds of flame when the initial pressures are 1 atm and 5 atm.

The sensitivity coefficients calculated for the NH_3_/Air flame and the CH_4_/NH_3_/Air flame at initial pressures of 1 atm and 5 atm, respectively, are as shown in Figure 12. As shown, R39 is the reaction with the highest sensitivity coefficient, which is much higher than that of the other reactions. The sensitivity coefficient of R39 decreases with the increase in pressure for the NH_3_/Air flame, but it increases for the CH_4_/NH_3_/Air flame. Other reactions that have a great influence on flame extinction have mixed changes when the pressure is 5 atm.

For instance, the sensitivity coefficients of R242 NH + NO<=>N_2_ + OH, R256 NH_2_ + NO<=>N_2_ + H_2_O, and R271 H + NO + M<=>HNO + M in the NH_3_/Air flame decrease with the increase in pressure, indicating that the inhibition effect of these reactions on pure ammonia flame decreases with the increase in pressure. Furthermore, the main inhibiting reaction switches from R273 HNO + H<=>H_2_ + NO to R88 with the rise of pressure, and the sensitivity coefficient of R88 is larger. The generation and consumption of HNO radicals no longer affect flame extinction. However, the generation (R36 H + O_2_ + H_2_O<=>HO_2_ + H_2_O) and consumption (R88) of inactive radicals, such as HO_2_, have a stronger promoting effect on flame extinction.

As can be seen in the CH_4_/NH_3_/Air flame, the inhibition ability of R36 increases, R88 switches from an inhibiting reaction to a promoting reaction, and R102 OH + CO<=>H + CO_2_ changes from a promoting reaction to an inhibiting reaction with the increase in initial pressure. The sensitivity coefficients of R85 and R278 in the two kinds of flame increased obviously. In addition, it can also be observed that when the pressure is 5 atm, the sensitivity coefficients of the reactions involving trisomy M in the two flames, such as the inhibiting reaction R44 H + OH + M<=>H_2_O + M and R53 H + CH_3_(M)<=>CH_4_(+M), are higher than those under normal pressures, while the promoting reaction R271 decreases. Therefore, the three-body reaction might lead to the flame being more likely to be extinguished with the increase in pressure.

It is worth mentioning that Li et al. [31] found that the three-body reaction H + OH + M<=>H_2_O + M (R44 in the Okafor mechanism in this paper) had a significant influence on the propagation speed of the laminar flame under high pressure by the method of sensitivity analysis, although it belongs to the chemical kinetic analysis of H_2_ combustion. However, there must be some commonality in the high-pressure combustion of NH_3_ fuel, since NH_3_ is the carrier of H_2_, which needs to be further analyzed and studied.

**Figure 13:**
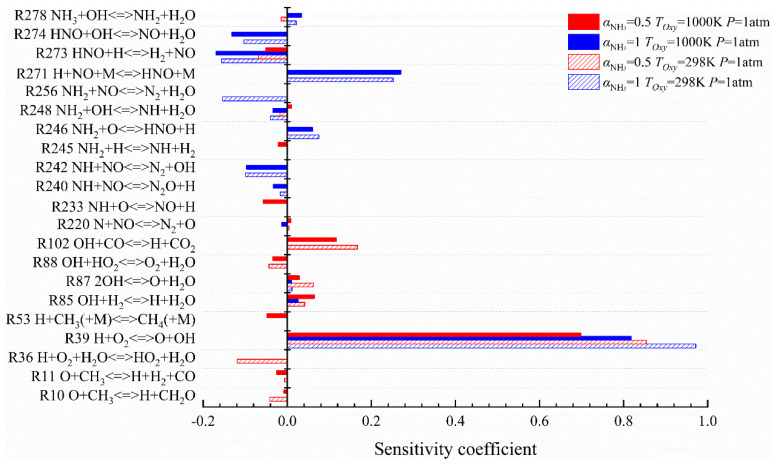
Corresponding reaction sensitivity coefficients of two kinds of flame when the air preheating temperatures are 298 K and 1000 K.

The sensitivity coefficients calculated for the NH_3_/Air flame and the CH_4_/NH_3_/Air flame at oxidant sides and an initial temperature of 298 K and 1000 K, respectively, were as shown in Figure 13. No matter how the initial conditions fluctuate, R39 is still the largest sensitivity coefficient response. R271 in the NH_3_/Air flame and R102 in the CH_4_/NH_3_/Air flame belong to the promoting reaction, whose sensitivity coefficient is second only to R39 in their respective flames. Additionally, both R85 and R87 are chain-branching reactions in active radical pools, and R220 N + NO<=>N_2_+O is the reaction of chain termination, which can facilitate the conduction of a branched-chain reaction and increase the flame extinction stretch rate in each flame.

As for the inhibiting reactions of the flames, the performance of the two kinds of flame is quite different, as outlined in the following description:

In the NH_3_/Air flame, R273 is the inhibiting reaction with the largest sensitivity coefficient, R256 is the second, and the rest of the reactions are as follows R240, R242, and R274, which are inhibiting reactions with small sensitivity coefficients. Furthermore, the promoting reactions R246 and R271 can generate HNO radicals, while the inhibiting reactions R273 and R274 consume HNO radicals. And, the absolute values of sensitivity coefficients of these four reactions are all second only to R39. Therefore, the existence of HNO is of critical importance in the continuous combustion when the flame is near the extinction limit, and the formation of HNO could promote the positive progress of the N2 chain when the flame is near the extinction limit, thus eventually increasing the extinction stretch rate.

In the CH_4_/NH_3_/Air flame, R273 is the inhibiting reaction whose sensitivity coefficient is second only to R36, promoting the flame extinction due to the consumption of HNO as well, but its sensitivity coefficient is far lower than that of the NH_3_/Air flame, indicating that the addition of CH_4_ would weaken the reaction intensity of each branch chain reaction in the N_2_ chain to a certain extent, and enhance the reaction intensity of each reaction in the chain containing C. The increase in air preheating temperature is capable of changing the dehydrogenation reaction R248 in the CH_4_/NH_3_/Air flame from an inhibiting reaction to a promoting reaction, and reducing the sensitivity coefficients of inhibiting reactions such as R10 O+CH_3_<=>H+CH_2_O, R88, and R271, which can generate the stable compound H_2_O and debilitate the inhibition effect of flame extinction so as to promote the positive progress of combustion.

## 3. Numerical Modeling and Research Methods

**Figure 14:**
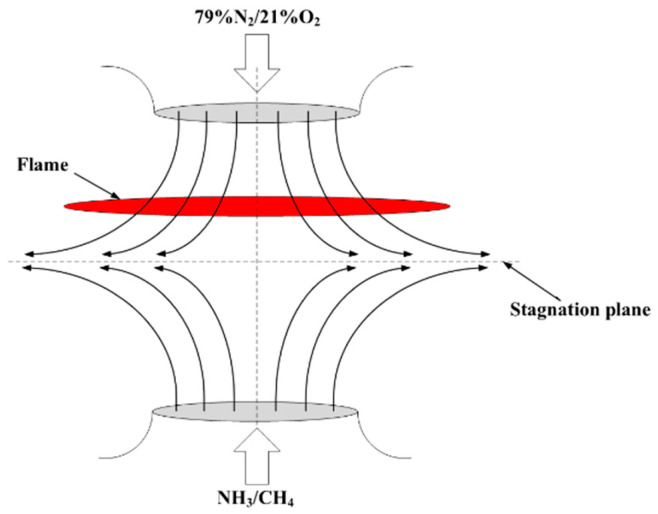
Geometric structure of laminar counterflow diffusion flame.

A numerical simulation for the opposed-flow flame was performed by using Chemkin-Pro software, which has already demonstrated the performance and utility of this method in previous studies of stretched flames [16,17,18]. Figure 14 shows the numerical model schematic of an axisymmetric non-premixed flame. It is observed that the configuration consists of two counterflow nozzles with the same axis. The stable counterflow diffusion flame is formed near the stagnation surface between the two nozzles. The relevant mathematical description of this physical model has been expressed in our previous study [18].

$\alpha_{{\mathrm{NH}}_{3}}$ is defined as the ammonia blending ratio, which is expressed as the mole fraction of NH_3_ in the NH_3_−CH_4_ binary fuel:

(3) $$\alpha_{{\mathrm{NH}}_{3}}=\frac{X_{{\mathrm{NH}}_{3}}}{X_{{\mathrm{NH}}_{3}}+X_{{\mathrm{CH}}_{4}}}$$

where $X_{{\mathrm{NH}}_{3}}$ and $X_{{\mathrm{CH}}_{4}}$ represent the mole fraction of NH_3_ and CH_4_, respectively. The range of ammonia blending ratio and the choice of chemical mechanism were determined based on the comparison of experimental data and numerical simulation results. Concerning mechanism verification, the related work has been completed by Colson et al. [32]. In his previous work, the extinction stretch rate of the CH_4_/NH_3_/Air counterflow diffusion flame with a wide range of ammonia mixing ratios was determined through experiments. The experimental results were compared with the numerical results simulated by four detailed chemical reaction mechanisms (the San Diego mechanism [33], Okafor mechanism [14], GRI Mech 3.0 [34], and Tian mechanism [35]). The results show that Okafor’s mechanism accurately predicted the extinction stretch rate in CH_4_/NH_3_/Air non-premixed flames with an ammonia content of 0–50%. In addition, the Okafor mechanism can perform remarkably well in the numerical simulation of other combustion characteristics of the CH_4_/NH_3_ mixed fuel, such as laminar flame propagation velocity [12] or Markstein length [14]. Therefore, the Okafor mechanism is chosen as the main detailed chemical reaction mechanism in the subsequent numerical calculation. Furthermore, the flame stretch rate *a* is defined by the classical formula calculated by Balakrishnan et al. [36] as follows:

(4) $$a=\frac{2\left(-V_{O}\right)}{L}\left[1+\frac{V_{F}}{\left(-V_{O}\right)}\sqrt{\frac{\rho_{F}}{\rho_{O}}}\right]$$

where *V_O_* and *V_F_* are the outlet flow velocities of the oxidizer side and fuel side nozzles, respectively, and *ρ_O_* and *ρ_F_* are the gas density of the oxidizer and fuel, respectively. *L* is the distance between two nozzles, which is 2 cm in this study. It can be observed from Figure 14 that all diffusion flames are biased towards the oxidant side. The momentum conservation equation is shown in Formula (5):

*ρ_F_V_F_*^2^ = *ρ_O_V_O_*^2^ (5)

Combining Equation (4) with (5), the relationship between the oxidant side flow rate and the fuel side flow rate can be deduced as follows:

(6) $$\left|V_{F}\right|=\left|V_{O}\right|\sqrt{\frac{\rho_{O}}{\rho_{F}}}$$

In the meantime, Formula (7) can be obtained through simultaneous Equations (5) and (6):

(7) $$\left|V_{O}\right|=\frac{aL}{4}$$

Through the above operation method, the preliminary calculation conditions can be obtained as shown in Table 7.

A path analysis and sensitivity analysis are also used in this study, which are briefly introduced below.

Path analysis is a method commonly used to analyze the chemical reaction mechanism in combustion chemical reactions, which can visually express the chain reaction as a form of component flow [37]. As a result, the chemical reaction in the combustion process can be accurately grasped. In the path analysis method, the rate of production or consumption of any component *c_j_* can be expressed by the following formula:

(8) $$\frac{dc_{j}}{dt}=v\frac{dc_{j}}{dx}=f_{j}\left(c,k\right)=\sum_{i=1}^{m}v_{ij}R_{i}(k_{i})$$

where $v_{ij}$ is the reaction term constant and *R_i_* is the chemical reaction rate of reaction *i*, which can be expressed by the following formula:

(9) $$R_{i}=k_{i}\prod_{j}^{n}\left\{c_{j}^{v_{ij}}\left|v_{ij}>0\right.\right\}$$

The contribution of arbitrary reaction *i* or component *k* to the formation of component *j* can be expressed as:

(10) $$N_{ij}=\frac{v_{ij}R_{i}(k_{i})}{\sum_{i=1}^{m}v_{ij}R_{i}(k_{i})}$$

(11) $$P_{ij}=\frac{k_{k}\prod_{j}^{n}\left\{c_{j}^{v_{ij}}\left|v_{ij}>0\right.\right\}}{\sum_{i=1}^{m}v_{ij}R_{i}(k_{i})}$$

Sensitivity analysis is a method used to systematically and quantitatively determine the relationship between the solution of the numerical model and the changes in related parameters. The effects of flame combustion characteristics such as radical production, flame temperature, heat release rate, laminar flame propagation velocity, or the extinction limit can be calculated by changing the pre-exponential factor *A* of the reaction. Some parameters that have a great or subtle influence on the solution can be obtained by a sensitivity analysis of the research object.

For the common steady-state combustion problems, the vector expression of the governing equations is as follows:

(12) F(ϕ(α),α)=0

where *F* is the residual of the equation, *α* is the parameter variable, and *φ* is the solution vector of the equation. By the differential operation of Equation (10) to *F*, the equation about the sensitivity coefficient can be obtained:

(13) $$\frac{\partial F}{\partial \phi }\frac{\partial \phi }{\partial \alpha }{|}_{F}+\frac{\partial F}{\partial \alpha }=0$$

where $\frac{\partial F}{\partial \phi }$ is the Jacobian matrix in the original system. $\frac{\partial F}{\partial \alpha }$ represents the partial derivative matrix of residual *F* to parameter *α*, each column of the matrix represents a functional relationship between a parameter and residual vector *F*, and quantitative information about how the parameter variables affect the solution can be obtained from this matrix, which is called the sensitivity coefficient.

In this study, the chemical kinetic characteristics of the NH_3_/Air flame and the CH_4_/NH_3_/Air counterflow diffusion flame with a 50% ammonia content under different initial temperatures or pressures were analyzed and discussed utilizing a path analysis and a sensitivity analysis. Furthermore, we endeavor to discover the free radicals and reactions that have a significant influence on the two kinds of flame extinction characteristics, as well as the specific effect of these reactions on flame extinction.

**Figure 15:**
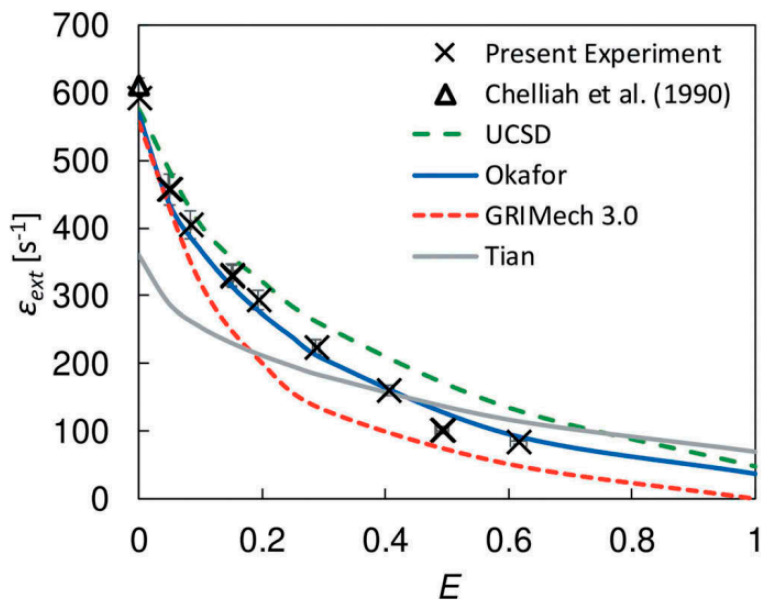
Extinction stretch rates of NH_3_/CH_4_/air non-premixed counterflow flame at different E values [32].

The choice of the chemical reaction mechanism was based on the Okafor mechanism, Tian mechanism, UCSD mechanism, and GRI-Mech 3.0 mechanism [32]. The comparison results are shown in Figure 15. The two solid lines and two dotted lines in the figure represent the Okafor mechanism, Tian mechanism, UCSD mechanism, and GRIMech 3.0 mechanism, respectively, and the scattered points represent the experimental values. The abscissa E is the calorific value fraction of ammonia in the fuel mixture, defined as the following equation:

(14) $$E=\frac{{\mathrm{LHV}}_{NH_{3}}\cdot X_{NH_{3}}}{{\mathrm{LHV}}_{NH_{3}}\cdot X_{NH_{3}}+{\mathrm{LHV}}_{CH_{4}}\cdot X_{CH_{4}}}$$

where $X_{NH_{3}}$ and $X_{{\mathrm{CH}}_{4}}$ represent the mole fraction of NH_3_ and CH_4_ in the NH_3_−CH_4_ binary fuel, respectively. LHV stands for low calorific value, and $LHV_{NH_{3}}=316.8\;kJ/kmol,\;LHV_{CH_{4}}=802.3\;kJ/kmol$.

It can be seen from the figure that the Okafor mechanism shows a perfect fitting effect on the extinction rate of the diffusion flame in all the current experimental conditions (E = 0~0.6), rather than the other three mechanisms. Compared with the experimental data, the relative error of the calculated results of the Okafor mechanism is also significantly smaller than that of the other three mechanisms. In addition, the Okafor mechanism can also perform very well in the numerical simulation of other flame characteristics of the NH_3_/CH_4_ mixed fuel, such as laminar flame propagation velocity and Markstein length. Therefore, the Okafor mechanism is chosen in the numerical simulation of the flame extinction limit of the laminar flow diffusion flame.

## 4. Conclusions

In this present study, the extinction and chemical kinetic characteristics of the CH_4_/NH_3_/Air impact diffusion flame under the conditions of different ammonia blending ratios, different initial pressures, and different oxidant side initial temperatures are studied by using Chemkin-Pro software. The conclusions are as follows:

(1) The increase in the ammonia blending ratio can reduce the flame extinction limit and narrow the combustible range. The issue can be effectively solved by increasing the pressure or the initial temperature of the oxidant, which is capable of significantly increasing the flame extinction stretch rate and enhancing the flame combustion stability. The ammonia fuel could undergo a phase change when the initial pressure exceeds 5 atm, which will cause the combustion process to become complex; thus, the trend of flame extinction stretch rate increment change is irregular. On the contrary, it is incapable of causing such a result by increasing the initial temperature on the oxidant side.
(2) The path diagram of the NH_3_/Air flame chain reaction, drawn by a path analysis, displays that there are three oxidation paths of ammonia. The generation and consumption of OH radicals are of considerable importance in NH_3_ consumption in the combustion process of pure NH_3_ fuel or the CH_4_/NH_3_ mixed fuel. The net reaction consumption velocity of OH increased obviously in the near extinction state with the addition of CH_4_ in the mixed fuel. Furthermore, it can significantly enhance the propagation characteristics of the CH_4_/NH_3_/Air flame at a low stretch rate by increasing the initial pressure or air preheating temperature on the oxidizer side; thus, NH_3_ in the mixed fuel could more fully participate in the chemical reaction with each active radical. The net reaction rate of OH chain branches in each reaction is substantially increased, which leads the flame intensity and extinction stretch rate to increase.
(3) The results of a reaction impact factor analysis describe that the increase in initial pressure or air preheating temperature can enhance the stretch rate bearing capacity of the flame and strengthen the combustion stability of the flame, which caused the flame to be difficult to extinguish. Moreover, the discussion of RIF can better verify the conclusions about the extinction characteristics that we have previously obtained.
(4) The computation results of a sensitivity analysis showed that R39 H+O_2_<=>O+OH is the most sensitive promoting reaction in the NH_3_/Air or the CH_4_/NH_3_/Air flame under continuous *P* = 5 atm and *T_Oxy_* = 1000 K conditions. It is worth noting that the sensitivity coefficient of R39 decreases with the increase in pressure for the NH_3_/Air flame, but increases for the CH_4_/NH_3_/Air flame. In addition, other reactions that have a significant effect on flame extinction have miscellaneous deviations when the pressure is 5 atm. Ultimately, this analysis demonstrates that the increase in pressure might have a significant effect on the three-body reaction in the flame. On the other hand, the increase in air preheating temperature is capable of changing the property of R248 NH_2_ + OH<=>NH + H_2_O in the CH_4_/NH_3_/Air flame from an inhibiting reaction to a promoting reaction, reducing the sensitivity coefficient of inhibiting reactions such as R10 O + CH_3_<=>H + CH_2_O, R88 OH + HO_2_<=>O_2_ + H_2_O, and R271 H + NO + M<=>HNO + M. Thus, the inhibition effect of flame extinction is weakened, and the positive progress of flame combustion is promoted.

## Figures and Tables

**Table 1:** The counterflow diffusion flame of pure NH_3_/Air (*T* = 298 K and *P* = 1 atm).

Number	Reaction Process	*A*
R39	H + O_2_<=>O + OH	1.04 × 10^14^
R85	OH + H_2_<=>H + H_2_O	2.16 × 10^8^
R87	2OH<=>O + H_2_O	3.57 × 10^4^
R240	NH + NO<=>N_2_O + H	2.90 × 10^14^
R242	NH + NO<=>N_2_ + OH	2.20 × 10^13^
R246	NH_2_ + O<=>HNO + H	6.60 × 10^13^
R248	NH_2_ + OH<=>NH + H_2_O	4.00 × 10^6^
R256	NH_2_ + NO<=>N_2_ + H_2_O	2.80 × 10^20^
R271	H + NO + M<=>HNO + M	4.48 × 10^19^
R273	HNO + H<=>H_2_ + NO	9.00 × 10^11^
R274	HNO + OH<=>NO + H_2_O	1.30 × 107^6^
R278	NH_3_ + OH<=>NH_2_ + H_2_O	2.00 × 10^6^

**Table 2:** The counterflow diffusion CH_4_/NH_3_/Air flame with ammonia blending ratio of 0.5 (*T* = 298 K and *P* = 1 atm).

Number	Reaction Process	*A*
R10	O + CH_3_<=>H + CH_2_O	5.06 × 10^13^
R11	O + CH_3_<=>H + H_2_ + CO	3.37 × 10^13^
R36	H+O_2_+H_2_O<=>HO_2_+H_2_O	1.13 × 10^19^
R39	H + O_2_<=>O + OH	1.04 × 10^14^
R85	OH + H_2_<=>H + H_2_O	2.16 × 10^8^
R87	2OH<=>O + H_2_O	3.57 × 10^4^
R88	OH + HO_2_<=>O_2_ + H_2_O	0.50 × 10^16^
R102	OH + CO<=>H + CO_2_	4.76 × 10^7^
R220	N + NO<=>N_2_ + O	2.70 × 10^13^
R248	NH_2_ + OH<=>NH + H_2_O	4.00 × 10^6^
R273	HNO + H<=>H_2_ + NO	9.00 × 10^11^
R278	NH_3_ + OH<=>NH_2_ + H_2_O	2.00 × 10^6^

**Table 3:** The counterflow diffusion flame of pure NH_3_/Air (*T* = 298 K and *P* = 5 atm).

Number	Reaction Process	*A*
R36	H + O_2_ + H_2_O<=>HO_2_ + H_2_O	1.13 × 10^19^
R39	H + O_2_<=>O + OH	1.04 × 10^14^
R85	OH + H_2_<=>H + H_2_O	2.16 × 10^8^
R87	2OH<=>O + H_2_O	3.57 × 10^4^
R88	OH + HO_2_<=>O_2_ + H_2_O	0.50 × 10^16^
R240	NH + NO<=>N_2_O + H	2.90 × 10^14^
R242	NH + NO<=>N_2_ + OH	2.20 × 10^13^
R248	NH_2_ + OH<=>NH + H_2_O	4.00 × 10^6^
R256	NH_2_ + NO<=>N_2_ + H_2_O	2.80 × 10^20^
R265	NNH + O_2_<=>N_2_ + HO_2_	2.00 × 10^14^
R271	H+NO + M<=>HNO + M	4.48 × 10^19^
R278	NH_3_ + OH<=>NH_2_ + H2O	2.00 × 10^6^

**Table 4:** The counterflow diffusion of the CH_4_/NH_3_/Air flame with an ammonia blending ratio of 0.5 (*T* = 298 K and *P* = 5 atm).

Number	Reaction Process	*A*
R36	H + O_2_ + H_2_O<=>HO_2_ + H_2_O	2.60 × 10^19^
R39	H + O_2_<=>O + OH	1.04 × 10^14^
R44	H + OH + M<=>H_2_O + M	2.20 × 10^22^
R53	H + CH_3_ (+M)<=>CH_4_ (+M)	1.39 × 10^16^
R85	OH + H_2_<=>H + H_2_O	2.16 × 10^8^
R86	2OH (+M)<=>H_2_O_2_ (+M)	7.40 × 10^13^
R87	2OH<=>O + H_2_O	3.57 × 10^4^
R88	OH + HO_2_<=>O_2_ + H_2_O	0.50 × 10^16^
R102	OH + CO<=>H + CO_2_	4.76 × 10^7^
R248	NH_2_ + OH<=>NH + H_2_O	4.00 × 10^6^
R271	H + NO + M<=>HNO + M	4.48 × 10^19^
R278	NH_3_ + OH<=>NH_2_ + H_2_O	2.00 × 10^6^

**Table 5:** The counterflow diffusion flame of pure NH_3_/Air (*T_Oxy_* = 1000 K and *P* = 1 atm).

Number	Reaction process	*A*
R39	H + O_2_<=>O + OH	1.04 × 10^14^
R85	OH + H_2_<=>H + H_2_O	2.16 × 10^8^
R87	2OH<=>O + H_2_O	3.57 × 10^4^
R220	N + NO<=>N_2_ + O	2.70 × 10^13^
R240	NH + NO<=>N_2_O + H	2.90 × 10^14^
R242	NH + NO<=>N_2_ + OH	2.20 × 10^13^
R246	NH_2_ + O<=>HNO + H	6.60 × 10^13^
R248	NH_2_ + OH<=>NH + H_2_O	4.00 × 10^6^
R271	H + NO + M<=>HNO + M	4.48 × 10^19^
R273	HNO + H<=>H_2_ + NO	9.00 × 10^11^
R274	HNO + OH<=>NO + H_2_O	1.30 × 10^7^
R278	NH_3_ + OH<=>NH_2_ + H_2_O	2.00 × 10^6^

**Table 6:** The counterflow diffusion of the CH_4_/NH_3_/Air flame with an ammonia blending ratio of 0.5 (*T_Oxy_* = 1000 K and *P* = 1 atm).

Number	Reaction process	*A*
R10	O + CH_3_<=>H + CH_2_O	5.06 × 10^13^
R11	O + CH_3_<=>H + H_2_ + CO	3.37 × 10^13^
R39	H + O_2_<=>O + OH	1.04 × 10^14^
R53	H + CH_3_ (+M)<=>CH_4_ (+M)	1.39 × 10^16^
R85	OH + H_2_<=>H + H_2_O	2.16 × 10^8^
R87	2OH<=>O + H_2_O	3.57 × 10^4^
R88	OH + HO_2_<=>O_2_ + H_2_O	0.50 × 10^16^
R102	OH + CO<=>H + CO_2_	4.76 × 10^7^
R220	N + NO<=>N_2_ + O	2.70 × 10^13^
R233	NH + O<=>NO + H	9.20 × 10^13^
R245	NH_2_ + H<=>NH + H_2_	7.20 × 10^05^
R248	NH_2_ + OH<=>NH + H_2_O	4.00 × 10^6^
R273	HNO + H<=>H_2_ + NO	9.00 × 10^11^
R278	NH_3_ + OH<=>NH_2_ + H_2_O	2.00 × 10^6^

**Table 7:** Mole fraction of the two nozzle sides of the NH_3_/CH_4_/Air counterflow diffusion flame.

*a* (s^−1^)	Fuel	Oxidizer	*ρ_f_* (g/L)	*ρ_O_* (g/L)	$V_{O}$ (cm/s)	$V_{F}$ (cm/s)
10	pureCH_4_	79%N_2_ + 21%O_2_	0.655	1.180	5	6.712
10	90%CH_4_ + 10%NH_3_	79%N_2_ + 21%O_2_	0.659	1.180	5	6.692
10	80%CH_4_ + 20%NH_3_	79%N_2_ + 21%O_2_	0.663	1.180	5	6.670
10	70%CH_4_ + 30%NH_3_	79%N_2_ + 21%O_2_	0.667	1.18	5	6.650
10	60%CH_4_ + 40%NH_3_	79%N_2_ + 21%O_2_	0.671	1.180	5	6.631
10	50%CH_4_ + 50%NH_3_	79%N_2_ + 21%O_2_	0.675	1.180	5	6.611
30	pureCH_4_	79%N_2_ + 21%O_2_	0.655	1.180	15	20.136
30	90%CH_4_ + 10%NH_3_	79%N_2_ + 21%O_2_	0.659	1.180	15	20.076
30	80%CH_4_ + 20%NH_3_	79%N_2_ + 21%O_2_	0.663	1.180	15	20.010
30	70%CH_4_ + 30%NH_3_	79%N_2_ + 21%O_2_	0.667	1.180	15	19.950
30	60%CH_4_ + 40%NH_3_	79%N_2_ + 21%O_2_	0.671	1.180	15	19.893
30	50%CH_4_ + 50%NH_3_	79%N_2_ + 21%O_2_	0.675	1.180	15	19.833
50	pureCH_4_	79%N_2_ + 21%O_2_	0.655	1.180	25	33.561
50	90%CH_4_ + 10%NH_3_	79%N_2_ + 21%O_2_	0.659	1.180	25	33.457
50	80%CH_4_ + 20%NH_3_	79%N_2_ + 21%O_2_	0.663	1.180	25	33.353
50	70%CH_4_ + 30%NH_3_	79%N_2_ + 21%O_2_	0.667	1.180	25	33.251
50	60%CH_4_ + 40%NH_3_	79%N_2_ + 21%O_2_	0.671	1.180	25	33.149
50	50%CH_4_ + 50%NH_3_	79%N_2_ + 21%O_2_	0.675	1.180	25	33.049

## Data Availability

Dataset available upon request from the author.
